# The role of Dichaete in transcriptional regulation during Drosophila embryonic development

**DOI:** 10.1186/1471-2164-14-861

**Published:** 2013-12-08

**Authors:** Jelena Aleksic, Enrico Ferrero, Bettina Fischer, Shih Pei Shen, Steven Russell

**Affiliations:** 1 Department of Genetics, University of Cambridge, Cambridge, UK; 2 Cambridge Systems Biology Centre, University of Cambridge, Cambridge, UK; 3 Present address: Wellcome Trust-Medical Research Council Cambridge Stem Cell Institute, Tennis Court Road, Cambridge CB2 1QR, UK; 4 Present address: Meridigen Biotech, Taipei 114, Taiwan

**Keywords:** Sox, Drosophila, Dichaete, Prospero, Nervous system, Neural stem cells

## Abstract

**Background:**

Group B Sox domain transcription factors play conserved roles in the specification and development of the nervous system in higher metazoans. However, we know comparatively little about how these transcription factors regulate gene expression, and the analysis of *Sox* gene function in vertebrates is confounded by functional compensation between three closely related family members. In *Drosophila*, only two group B *Sox* genes, *Dichaete* and *SoxN*, have been shown to function during embryonic CNS development, providing a simpler system for understanding the functions of this important class of regulators.

**Results:**

Using a combination of transcriptional profiling and genome-wide binding analysis we conservatively identify over 1000 high confidence direct Dichaete target genes in the *Drosophila* genome. We show that Dichaete plays key roles in CNS development, regulating aspects of the temporal transcription factor sequence that confer neuroblast identity. Dichaete also shows a complex interaction with Prospero in the pathway controlling the switch from stem cell self-renewal to neural differentiation. Dichaete potentially regulates many more genes in the *Drosophila* genome and was found to be associated with over 2000 mapped regulatory elements.

**Conclusions:**

Our analysis suggests that Dichaete acts as a transcriptional hub, controlling multiple regulatory pathways during CNS development. These include a set of core CNS expressed genes that are also bound by the related Sox2 gene during mammalian CNS development. Furthermore, we identify Dichaete as one of the transcription factors involved in the neural stem cell transcriptional network, with evidence supporting the view that Dichaete is involved in controlling the temporal series of divisions regulating neuroblast identity.

## Background

Sox-box transcription factors play critical roles in the embryonic development of all metazoans in which they have been characterised [1]. The group B subgroup is of particular interest in vertebrate biology since it encodes a set of transcriptional regulators known to play critical roles in neural development and in the maintenance of embryonic stem cells [2-5]. In most vertebrates there are 5 group B genes: the group B1 genes *Sox1, 2* and *3*, which primarily act as transcriptional activators, and the group B2 genes *Sox14* and *21*, which act mainly as transcriptional repressors and appear to antagonise group B1 functions [6,7].

In *Drosophila* there are four group B genes; *SoxNeuro* (*SoxN*)*, Dichaete, Sox21a* and *Sox21b*, the latter three clustered in the genome in a complex found in all insect genomes so far sequenced [8-10]. While there is a lack of clarity regarding the subdivision of group B proteins in insects [9,11], it is clear that, as with vertebrate group B1 proteins, both Dichaete and SoxN play critical roles in the early development of the embryonic nervous system. The functions of *Sox21a* and *Sox21b* are currently not known. However, neither gene is expressed in the embryonic CNS until very late in development, suggesting that in the fly, only two group B genes are involved in early CNS development. Functional studies using mouse *Sox* B1 genes to rescue fly mutant phenotypes indicate that the mammalian proteins are functionally related to both *Dichaete* and *SoxN*[12,13]. Sox2 in particular was able to rescue Dichaete functions, suggesting a high degree of functional conservation. The study of Dichaete may therefore provide useful insights into aspects of Sox2 function in the more complex vertebrate CNS.

*Dichaete* is expressed dynamically during embryogenesis where it is known to have functions in early segmentation, CNS development, hindgut morphogenesis and cuticle patterning [14-17]. In the CNS, *Dichaete* is expressed early in the ventral midline, the fly equivalent of the vertebrate floor plate, as well as the medial and intermediate columns of the neuroectoderm from the earliest stages of CNS specification. *SoxN* is also expressed in the neuroectoderm, in this case extending more laterally to include the lateral column, and is not expressed in the midline until the latter half of embryogenesis [18]. Thus, as is the case with group B genes in vertebrates, both Dichaete and SoxN are co-expressed in many of the cells of the early CNS and they exhibit extensive functional compensation with double mutants exhibiting severe neural hypoplasia in contrast to the comparatively mild phenotypes of single mutants [19,20]. Targeted expression of dominant negative forms of Dichaete or its mammalian orthologue Sox2 also show strong CNS phenotypes, further demonstrating that group B function is critical for normal CNS development [21].

At the molecular level, both Dichaete and SoxN are required for the correct expression of pro-neural genes in the Achaete-scute Complex, particularly *achaete* (*ac*) and *asense* (*ase*), where they interact with the homeodomain proteins encoded by *intermediate neuroblasts defective* (*ind*) and *ventral neuroblasts defective* (*vnd*) [19,21-23]. Group B Sox proteins in the vertebrate CNS are also coexpressed with related homeodomain proteins along the ventro-medial axis of the developing neural tube, hinting at a deep conservation of Sox function in the CNS [24]. A role for Dichaete in the control of midline expression of the *slit* gene has been well characterised, with both genetic and molecular evidence demonstrating a critical interaction between Dichaete and the POU-domain protein Ventral veins lacking (Vvl) [13,25]. Mammalian Sox2 interacts with a related POU protein, Oct4, in the regulatory network controlling embryonic stem cell pluripotency and in primary neurogenesis [26-30]. Mouse Sox2 can also interact with Vvl in the fly, reinforcing the idea that Dichaete and Sox2 are functionally related [13].

More recently, a genomic analysis of embryos expressing dominant negative forms of Dichaete in combination with Dichaete binding data identified hundreds of potential target genes with annotated CNS functions, suggesting the *Drosophila* group B Sox proteins have widespread roles in CNS development [21]. Other studies have identified roles for Dichaete and SoxN in the regulation of *shavenbaby* in the epidermis, in part by repressing Wnt signalling through an interaction with the related HMG-domain protein Lef1/Tcf [17,31]. Finally, more recent work in the embryonic and larval CNS has shown that Dichaete plays a role in the transcriptional hierarchy that controls the temporal specification of neuroblast fate, in particular controlling the decisions to terminate the self-renewal program and differentiate or die [32]. This bears a striking resemblance to the role group B1 Sox proteins play in the vertebrate neuroepithelium, where their down-regulation is required for cell-cycle exit and subsequent neural differentiation [33].

In this study, we use genomics approaches, including DamID and transcriptional profiling, to gain insight into the role of Dichaete during embryonic CNS development. We also draw on previously published embryonic binding data generated by BDTNP [34] and the modENCODE project [35] to perform an integrative analysis of Dichaete function. We find that potential target genes are mainly downregulated in a *Dichaete* mutant, suggesting it may function largely as a transcriptional activator, and that Dichaete is one of the key factors in the *Drosophila* neural stem cell transcriptional network interacting with a temporal series of transcription factors regulating neuroblast fate. We also find evidence of an interaction with Prospero, which is apparently antagonistic in neuroblasts, but synergistic in the Ganglion Mother Cells. Together our findings support the view that Dichaete functionally acts as a group B1 Sox factor and further emphasises the striking conservation in Sox function in the CNS.

## Results

### Generating a core set of Dichaete binding intervals

Dichaete is important for a number of key developmental processes, including embryonic segmentation, hindgut morphogenesis and nervous system development. However, a thorough genomic analysis of the role of Dichaete during early *Drosophila* embryogenesis aimed at identifying target genes and regulatory networks has not been performed to date. Genome-wide binding data from blastoderm embryos is available from the Berkeley Drosophila Transcriptional Network Project [34] and data from later stages of embryogenesis was generated as part of the modENCODE project [35]. Our preliminary analysis indicated some discordance between these datasets. While some differences are to be expected due to different developmental stages used, the modENCODE data appeared to have weaker signal, and identified considerably fewer peaks than the DBTNP data. Since it was unclear whether this was due to false positives in the BDTNP data or false negatives in the modENCODE data, we elected to generate an independent genome-wide Dichaete binding dataset to identify high-confidence binding intervals supported by different analytical methods.

We used the DamID method [36] to generate Dichaete-Dam and control Dam profiles from three independent biological replicates of stage 5–11 embryos. After normalisation and peak-calling we found that Dichaete, in common with other transcription factors, is associated with thousands of genomic regions in the embryo across development. We identified 6067 binding intervals at 1% FDR and over 16,000 at 25% FDR. We assessed enrichment for know transcription factor binding motifs within the 1% FDR binding intervals using i-*cis*Target [37] and identified a Vielfaltig motif (Vfl; E-score = 6.2) [38] followed by a series of Sox binding motifs (E-scores from 4.5 to 5.1), suggesting these DamID data identify *bona fide* Dichaete genomic occupancy. Assigning the 1% FDR binding intervals to genes, we found Dichaete associated with 3561 genes (Additional file 1: Table S1). This Dichaete-bound set of genes is significantly enriched for general developmental and regulation terms (p < 1E-50), and also more specifically for nervous system development (581 genes, p = 9.2E-31), neuron generation (374 genes, p = 2.9E-42) and neuron differentiation (336 genes, p = 5.9E-35). We also found enrichment for genes involved in brain development (69 genes, p = 1.6E-7), hindgut morphogenesis (39 genes, p = 2.6E-4) and segmentation (122 genes, p = 1.4E-10), all processes Dichaete is known to be involved in (the full set of enrichments is given in Additional file 2: Table S2).

**Figure 1:**
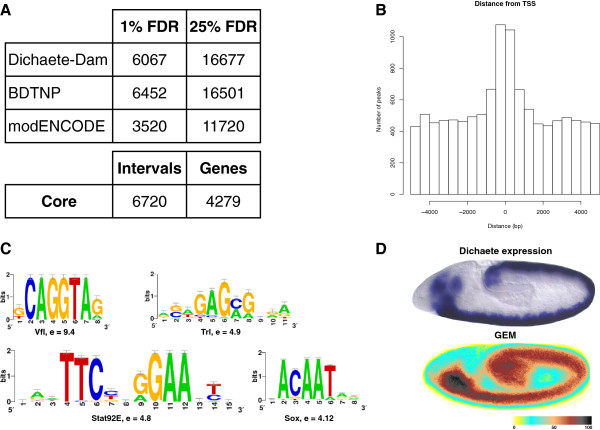
**Dichaete binding overview. A)** Number of binding intervals identified at different false discovery rates in the Dichaete DamID (stage 5–11), Berkeley (BDTNP; stage 4–5) and modENCODE (Stage 0–11; modENCODE_2571) datasets. The number of binding intervals and associated genes defined in the Dichaete core binding set. **B)** The distance of Dichaete peaks from transcription start sites, showing binding preference to the region within +/− 500 bp of a TSS. **C)** Top 4 enriched binding motifs in the Dichaete core intervals identified by i-*Cis*Target, including their e-scores. **D)** Top: lateral view of Dichaete *in situ* hybridisation in a stage 10 embryo. Bottom: Genome-wide Expression Map generated from the FlyExpress database from the Dichaete bound and regulated gene set.

The published ChIP datasets also identified large numbers of binding intervals and associated genes (BDTNP = 6452 binding intervals and modENCODE = 3520 binding intervals at FDR1%, mapping to approximately 6500 and 3500 genes, respectively). To focus on a set of high confidence Dichaete target genes and eliminate technique specific artefacts as far as possible, we combined our DamID dataset with the ChIP datasets to generate a set of core binding intervals. We identified intervals supported by at least one low stringency (25% FDR) and one high stringency (1% FDR) dataset from the independent experimental techniques; for example, all regions confirmed with 1% FDR DamID and 25% FDR ChIP were included, and vice versa (Figure 1A; Additional file 3: Table S3). Thus each of the intervals in the core dataset was supported by high stringency evidence in at least one dataset, and was also independently validated using an alternative experimental technique. These binding intervals were used for further analysis. While we recognise that this is a relatively stringent filter and will not include many *bona fide* Dichaete binding intervals, we contend that focusing on independently verified binding locations will allow us to make more confident inferences about Dichaete function in the embryo.

This amalgamation process generated a dataset of 6720 intervals associated with 4279 *Drosophila* genes (Additional file 4: Table S4), which were examined for general properties using the FlyMine data warehouse [39]. We found that almost half these genes (1925) are expressed in the larval CNS, an encouraging observation given the known role of Dichaete in larval neuroblasts [32]. We also found even stronger enrichment for genes associated with nervous system development (775 genes, p = 1.4E-67), neuron generation (461 genes, p = 2.1E-60), brain development (91 genes, p = 2.4E-17) and other nervous system terms (Additional file 5: Table S5). A large number of the Dichaete-bound genes have a role in the regulation of transcription (451 genes, 1.3E-47), with as many as two thirds (205 out of 294) of the FlyTF curated ‘trusted’ transcription factors featured in the gene list [40]. We also noticed a very strong enrichment for Dichaete binding at genes encoding specific transcription factor classes, including homeodomain proteins (99 homeodomain or homeodomain-like proteins, 3.0E-16); C2H2-type Zinc finger domain proteins (139 genes, 4.8E-5) and, to a lesser extent, fork head domain proteins (15 genes, 1.2E-2). Taken together, these data support the idea that Dichaete is a key developmental regulator controlling a battery of transcription factor genes involved in nervous system development and a variety of other developmental processes.

We examined the overall genomic distribution of the Dichaete core binding intervals, using the centre coordinate of each interval as an approximation of the Dichaete binding site. We found that two thirds of Dichaete binding (67%) maps to genic regions and of these, the majority (65%) are in introns. We also found an association between Dichaete binding and transcription start sites (TSS), observing a peak of binding intervals within 500 bp of a TSS, indicating a preference for this region (Figure 1B). However, this is a relatively weak predictor of overall Dichaete binding, with slightly less than 25% (1618 out of 6720) of the total Dichaete binding events found close to a TSS. We also found that Dichaete binding showed a significant preference for long introns (p-value < 2.2e-16, Wilcoxon rank sum test). The mean length of Dichaete-bound introns is 12.7 kb, while the mean length of all *Drosophila* introns excluding those bound by Dichaete is 1.5 kb (the median, which is less sensitive to outliers, still showed this difference: median Dichaete bound = 5.7 kb compared to 130 bp for the rest of the genome). While it is possible this observation is an artefact of larger regions of the genome being more likely to be bound in general, the high level of significance suggests this observation may well reflect an aspect of Dichaete function. Recent work does suggest that long and short introns are functionally distinct [41], with long introns being, for example, more likely to harbour regulatory sequences.

The Dichaete core dataset was also found to overlap with a sizeable proportion of mapped enhancer regions. Of a total of 8975 reported enhancers (1862 from RedFly and 7113 from FlyLight), Dichaete binding was associated with 2400 (27%), including almost half of the experimentally validated enhancers reported in RedFly (887 out of 1862) [42]. The 887 Dichaete-bound RedFly enhancers are associated with a total of 229 genes, including *vvl*, encoding a known Dichaete midline cofactor, the neuroectoderm column specifying gene *vnd*, as well as genes for transcription factors with roles in neuroblast pluripotency and differentiation such as *hb*, *Kr*, *nub*, *grh* and *mira*[43,44]. Substantial overlap was found with the enhancers mapped by the FlyLight project [45,46], particularly with those showing expression in neuroblasts (28/30), the midline (114/201 early and 476/1072 late) and in large subsets of the CNS (478/811) (Additional file 6: Table S6). Together these data indicate that Dichaete associates with both core promoter regions and with classical enhancers.

As noted above, our DamID dataset was enriched for Vfl and Sox-related binding motifs. A similar analysis with the BDTNP and modENCODE binding intervals again identified strong enrichment for Vfl motifs (E-scores of 9.4 and 8.1 respectively) and weaker enrichment for Sox-like motifs (E-scores 2.6 and 2.8). In our core dataset the top enriched motifs were Vfl (E-score 9.4), Trithorax-like (Trl, E-score 4.9), Stat92E (E-score 4.8) and Sox motifs (E-score 4.1) (Figure 1C). Using a previously published Dichaete motif from a bacterial 1-hybrid screen for a motif scan [47], we found that 82% of all intervals contain matches to the motif, supporting the view that the core binding intervals represent specific Dichaete binding sites.

Taken together, these observations suggest a role for Dichaete in the transcriptional regulatory network active in the developing CNS, including its previously reported function in the midline, as well as a role in neuroblasts. Binding associated with hundreds of mapped enhancer regions and an association with transcription start sites indicates that Dichaete may display two distinct modes of operation at enhancers and promoters. Alternatively, this may reflect aspects of higher order chromatin organisation where Dichaete bound enhancers are brought into close proximity to core promoter regions.

### Dichaete may act as a regulatory hub during embryogenesis

It is known that essential proteins in densely interconnected biological processes such as development are more likely to be genetic network hubs - regulators connected to a large number of network nodes [48,49]. A number of developmental transcription factors fit this role, and between themselves these also tend to be highly interconnected. In the case of Dichaete, the large number of binding regions in the genome suggested a potential role as a hub, supported by the finding that the binding is frequently associated with other transcriptional regulators. To investigate whether Dichaete does indeed appear to have a regulatory role for a large number of targets, we performed a gene expression analysis in *Dichaete* mutant null embryos.

We have previously performed targeted gene expression analysis of group B Sox function in the CNS using dominant negative constructs [21]. To gain a more comprehensive view of Dichaete function during development we performed microarray expression profiling with RNA extracted from *Dichaete* mutants. We isolated RNA from stage 10–11 *Dichaete* null mutant embryos and identified genes with significantly changed expression compared to heterozygous siblings. Remarkably, we found that the majority of genes in the genome appeared to change expression and consequently our usual data normalization methods were inadequate. We therefore utilised a normalization approach that relied on identifying the minority of invariant genes and using these to normalise the remainder [50]. With moderate stringency statistical thresholds (M-value > 0.5 or < −0.5, p-value < 0.05), 9120 genes were differentially expressed: approximately 65% of the genes in the *Drosophila* genome. In order to focus on a reliable set of differentially expressed genes, we applied a more stringent threshold (M-value > 1 or < −1, p-value < 0.01), identifying 4518 differentially expressed genes, and these were used for further analysis (Additional file 7: Table S7). The vast majority of these (>90%, 4182 genes) were found to be downregulated, with only 336 genes upregulated in the mutant. While some of these gene expression changes are likely to be pleiotropic effects reflecting the early role of Dichaete in segmentation, encouragingly we still found significant enrichment for genes involved in development (p = 7.2E-9) and neurogenesis (252 genes, p = 5.3E-6) and submitting the gene list to the FlyExpress database generated a Genome-wide Expression Map that highlights the developing CNS, supporting the view that *Dichaete* regulates genes in the CNS (Figure 1D) [51]. While there is some overlap between genes involved in segmentation and those involved in neurogenesis, segmentation genes alone do not account for all the neurogenesis genes affected, and pleiotropy is unlikely to explain all the changes we observe. Previous studies have found that regions of the embryo such as the thorax, which are largely unaffected by the segmentation phenotype, still display strong neural phenotypes [13]. Furthermore, at the level of the individual embryo, segmentation defects are highly variable with many individuals showing comparatively weak phenotypes [14,15]. In contrast, we focus here on very strong changes in gene expression that are likely to reflect more widespread consequences of *Dichaete* loss. Although we identify a strong CNS signature in the GO enrichments, the most enriched GO term in these data was for Proteolysis (293 genes, p = 1.2E-10), which may possibly reflect a generic stress response, suggesting that many of the identified genes may change expression as an indirect response to the loss of Dichaete function.

To focus on likely direct target genes we intersected the core binding intervals with the mutant gene expression data. This generated a set of 1373 genes that show significant expression changes in *Dichaete* mutant embryos and are bound by Dichaete in independent binding studies: we refer to these as Dichaete target genes (Additional file 8: Table S8, which for reference also includes previously reported expression in dominant negatives [21]). We emphasise that both the binding and the expression data were analysed with relatively stringent cut-offs and that the true number of Dichaete targets is likely to be higher. The Dichaete target genes are highly enriched for developmental processes (3E-54), and nervous system specific annotations (generation of neurons, p = 2.4E-39; nervous system development, p = 1.4E-32; neuron differentiation, p = 1.8E-33) (Additional file 9: Table S9). Again we found the list of targets was strongly enriched for transcription factors, with approximately half (152) of the 296 genes present in the FlyTF ‘trusted’ list of transcription factors [40] differentially expressed in the Dichaete null mutant. This was reflected in enrichment for gene transcription/expression GO terms (p = 1.3E-34), and for genes encoding Homeodomain (50 genes, p = 5.8E-18), fork head (12 genes p = 2.1E-4) and C2H2 Zinc finger (65 genes, p = 2.1E-4) genes. These high-confidence Dichaete target genes further support the view that Dichaete plays a key role in embryonic development and suggests it may act as a regulatory hub.

### Dichaete in the hindgut

**Figure 2:**
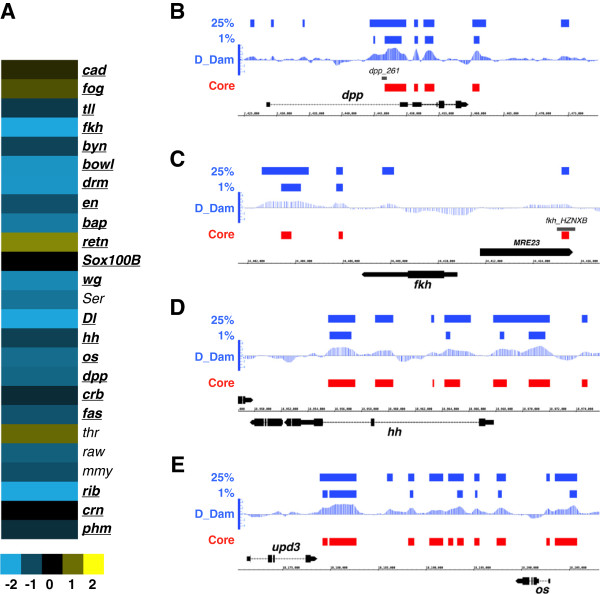
**Dichaete in the hindgut. A)** Expression levels of hindgut expressed genes in *Dichaete* mutants with genes ordered according to their position in the regulatory hierarchy. Scale bar represents log2 expression ratio. **B – E)** Dichaete binding profiles at indicated hindgut genes. Upper two blue plots represent 25% and 1% FDR DamID binding intervals; D_Dam represents the normalised window score of the triplicated DamID experiment; Core (red) represents the regions defined as core binding intervals overlapping DamID and ChIP data. The black regions represent gene models and the genome coordinates. The named grey bars in B and C represent mapped regulatory regions.

We have previously shown that Dichaete is required for correct morphogenesis of the embryonic hindgut, where it positively regulates *dpp* and negatively regulates *hh*[16]. We examined the Dichaete binding and expression data to determine the extent of Dichaete involvement in gut development. We found many of the genes known to be involved in aspects of hindgut development were bound and apparently controlled by Dichaete. Hindgut development is modulated by a cascade of transcription factors and signalling pathways, starting with the activity of the terminal genes *tll* and *hkb*, through a highly conserved “gastrulation cassette” (*cad, byn, fkh* and *wg*) to a set of patterning and morphogenesis genes (*bowl, dpp, drm, en, hh, lin, upd* and *Dichaete* itself) [52]. Of 25 genes known to be involved in hindgut development, we found that 17 were strongly downregulated in Dichaete mutant embryos and three showed weak upregulation (Figure 2). While *hh* is repressed by Dichaete in the hindgut, our expression profiling shows downregulation of *hh* in *Dichaete* mutants, this is likely a reflection of loss of *hh* expression in the CNS. In addition, we observed Dichaete core binding intervals associated with 21 of these genes, including at sites overlapping known hindgut enhancer elements in the *dpp* and *fkh* genes as well as enhancers showing embryonic gut expression in the FlyLight collection (*dpp, hh, wg, retn, crb*) [45,46] (Figure 2).

### Known Dichaete targets in the CNS

**Figure 3:**
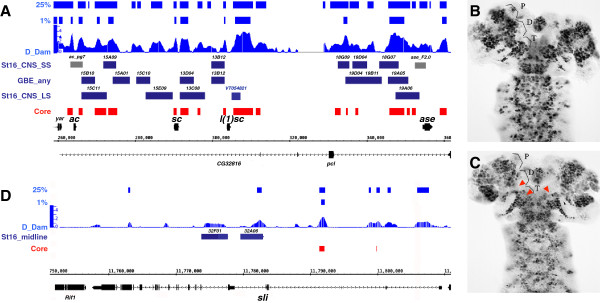
**Dichaete at known target genes. A)** Achaete-scute Complex: Dichaete Dam ID binding profiles with 1% and 25% FDR intervals. Dark blue tracks represent FlyLight Enhancers with expression categorised as; small subset of the CNS at stage 16 (st16_CNS_SS), any CNS in germ band extended embryos (GBE) and large subset of the CNS at stage 16 (St16_CNS_LS). Grey bars represent other mapped enhancer elements. Core (red) represents the regions defined as core binding intervals overlapping DamID and ChIP data. The black regions represent gene models and the genome coordinates. **B** and **C)** antibody staining revealing *l(1)sc* expression in wild type **(B)** and *D*^*r8*^*/Df(3 L)GS1-a***(C)** stage 16 embryos. P, D and T refer to the protocerebrum, deutocerebrum and tritocerebrum. Red arrowheads indicate loss of *l(1)sc* expression in the duetocerebrum and tritocerebrum. **D)***slit* tracks as above with FlyLight enhancers identified as expressed in the midline at stage 16.

Previous work has shown that Dichaete directly regulates genes of the *AS-C* in the neuroectoderm and *sli* in the midline [21,23,25] and we found all four genes in the complex downregulated at least 2-fold in *Dichaete* mutants. We identified substantial Dichaete binding across the *AS-C* and almost all of the core binding intervals in this region overlap with known RedFly enhancers (*ac_pg7* and *ase_F2.0*) or regulatory elements identified by the FlyLight project [45,46] (Figure 3A). We also noticed a prominent binding interval 3′ to *l(1)sc*, where no embryonic regulatory regions are reported in the FlyLight set, however there is a VT enhancer region reported here [53].

While Dichaete has been shown to affect the expression of *ac, sc* and *ase*, there have been no reports relating to *l(1)sc* in *Dichaete* mutants: while we find that ventral nerve cord *l(1)*sc expression is apparently unaffected in *Dichaete* null mutant embryos, we noticed a loss of expression in specific populations of cells in the deuteocerebral and protcerebral regions of the embryonic brain (Figure 3B-C), [54]). Together these observations strongly suggest that Dichaete interacts with many of the known regulatory elements in the *AS-C* and that at least some of this binding is functionally relevant to embryonic expression of the proneural genes in the Complex.

In the case of *sli*, which Dichaete regulates in midline glia, we find some evidence of Dichaete binding, however, most of the intervals are not identified in the core set, presumably because midline glia comprise a very small fraction of the whole embryo. Previous work showed that Dichaete binding can be detected at the midline enhancer with specific PCR assays despite an apparent lack of binding in BDTNP and modENCODE datasets [21,25] and we note that a low stringency DamID peak overlaps the *GMR32A06* regulatory region encompassing the *sli* midline enhancer. There is a core Dichaete binding interval within the 1^st^*sli* intron, where a regulatory element controlling expression in the posterior gut is reported [55], and *sli* expression is downregulated in Dichaete mutants (2.6 fold). Thus our data support the view that Dichaete more broadly regulates *sli* expression (Figure 3D).

### Dichaete in the neuroectoderm

**Figure 4:**
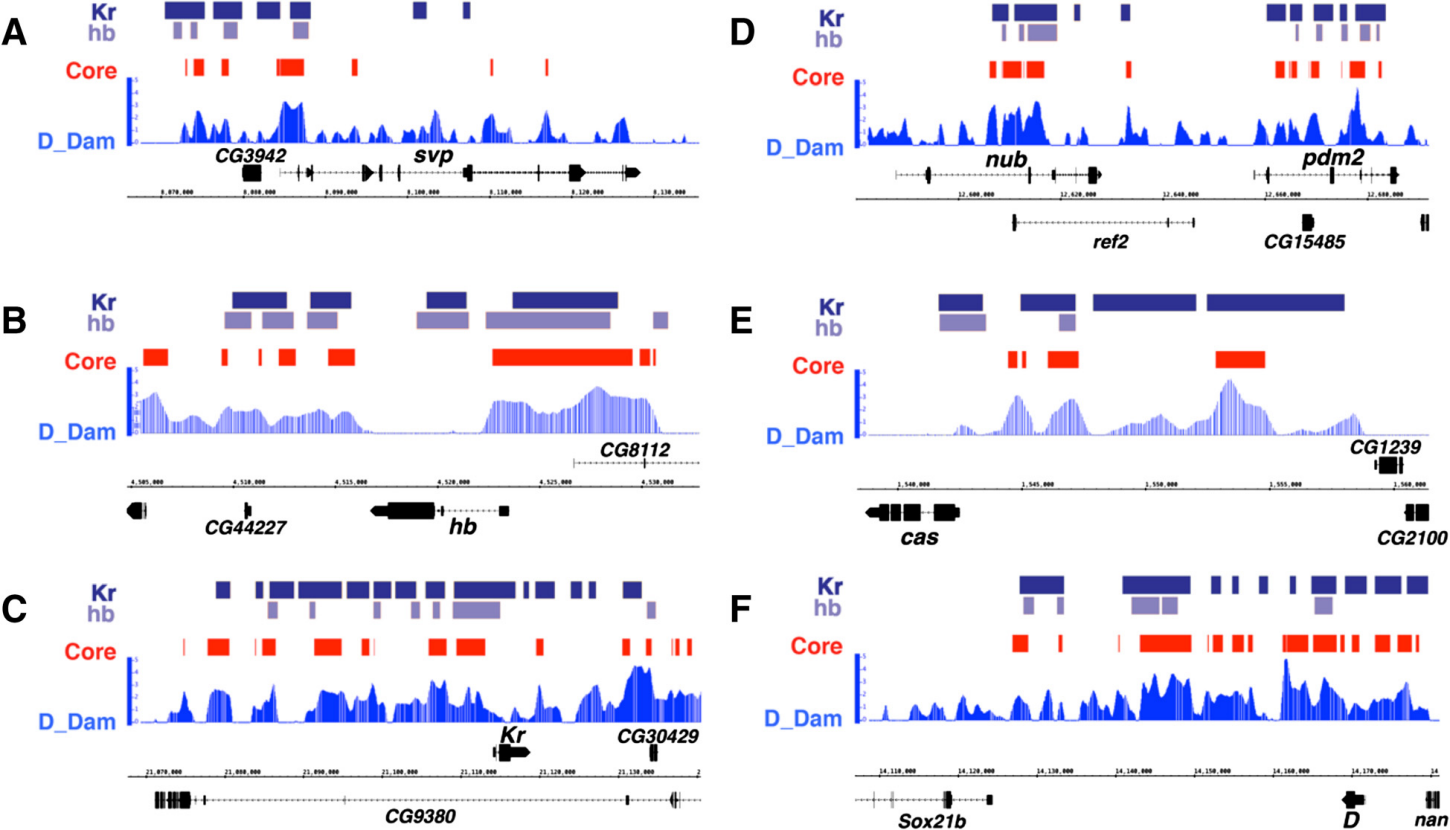
**Temporal neuroblast cascade. A-F)** Dichaete DamID profiles (blue) and core binding intervals (red) along with the 1% FDR binding intervals for Kr (dark blue) and Hb (light blue) from the BDTNP at the 5 genes of the temporal neuroblast cascade and the *Dichaete* region.

Dichaete is dynamically expressed in the neuroectoderm, in neuroblasts, GMCs and in differentiating neural cells, and our Dichaete target gene set strongly suggests that it regulates several hundred genes in these lineages. In contrast, *Dichaete* mutant phenotypes are relatively weak in the ventral nerve cord due to functional compensation by the related group B gene *SoxN*. To gain insights into Dichaete function in the ventral nerve cord we focused our attentions on some of the core regulatory pathways controlling neural differentiation. In the embryonic and larval CNS, Dichaete has been reported to play a role in regulating the switch from neuroblast self-renewal to cell cycle exit and differentiation or apoptosis [32]. We therefore examined the Dichaete target set to seek evidence for similar activity in the embryonic nervous system. Strikingly, we found extensive binding at all of the genes implicated in the temporal transcription factor cascade (*svp, hb, Kr, nub, pdm2* and *cas*) with most of these downregulated more than 2-fold in *Dichaete* mutants (*hb* and *nub* were downregulated approximately 1.5-fold). There are cross-regulatory interactions between the transcription factors governing neuroblast fate and we therefore compared Dichaete core binding with available data for Hb and Kr. We found extensive overlap between Dichaete core binding intervals and the high stringency (1% FDR) Kr and Hb binding intervals determined by BDTNP at all of the core temporal transcription factors (Figure 4). Although there are no available binding data for Svp, Nub/Pdm2 or Cas, a genetic interaction between Nub/Pdm2 and Dichaete has been reported [56].

**Figure 5:**
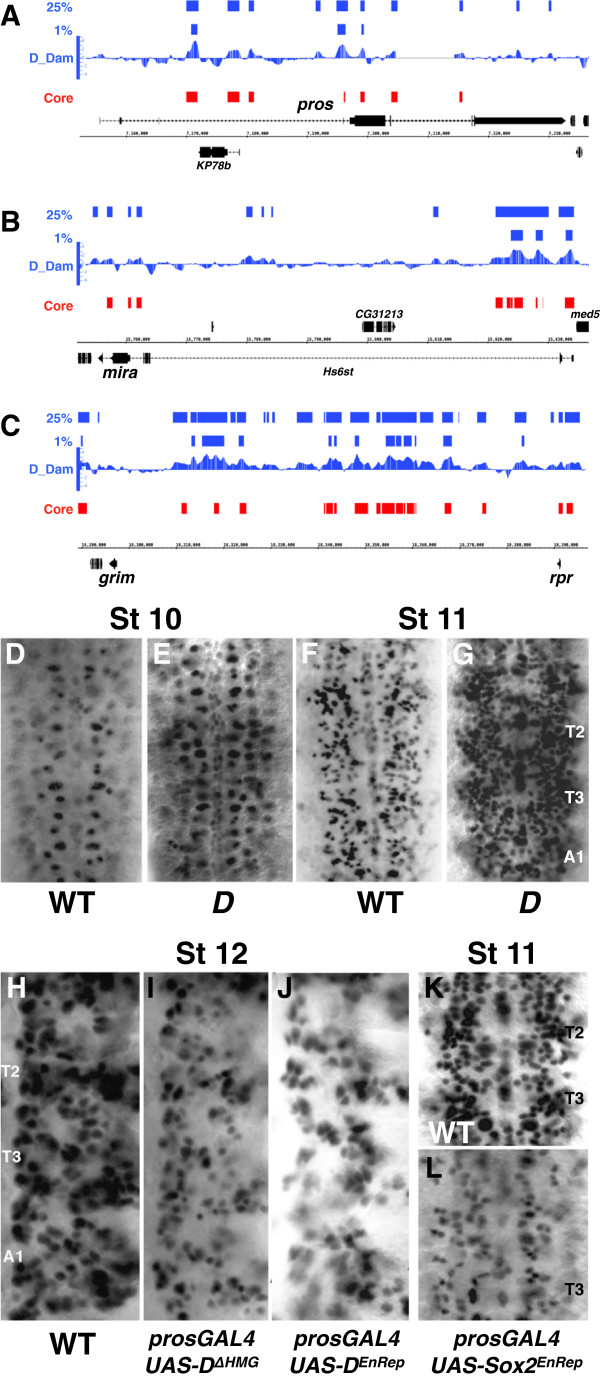
**Dichaete and neuroblast segregation. A-C)** Dichaete binding profiles at *pros, mira* and *grim-rpr*. Upper two blue plots represent 25% and 1% FDR DamID binding intervals; D_Dam represents the normalised window score of the triplicated DamID experiment; Core (red) represents the regions defined as core binding intervals overlapping DamID and ChIP data. The black regions represent gene models and the genome coordinates. **D-G)** anti-Prospero staining in *Dichaete* mutant embryos, all ventral view with anterior to the top. **D)** wild type stage 10. **E)***D*^*r72*^*/Df(3 L)Gs1-a* stage 10. **F)** wild type stage 11. **G)***D*^*r72*^*/Df(3 L)Gs1-a* stage 11. **H-J)** Anti-Prospero staining in embryos expressing dominant negative Dichaete constructs, images show the right half of two thoracic and 1 abdominal segment (T2, T3, A1). **H)** wild type, stage 12. **I)***prosGAL4, UAS-D*^*ΔHMG*^ stage 12. **J)***prosGAL4, UAS-D*^*EnRep*^ stage 12. **K** and **L)** Anti-Pros staining in embryos expressing dominant negative mouse Sox2, ventral views of 2 abdominal segments from late stage 11 embryos. **K)** wild type. **L)***prosGAL4, UAS-mouseSox2*^*EnRep*^.

The termination of the neuroblast programme is marked by the segregation of Prospero and Miranda to the GMC, the nuclear localisation of Prospero and the decision to differentiate or die. We found extensive Dichaete binding across the *pros* gene (Figure 5A), and Dichaete core binding associated with *mira* (Figure 5B) and *insc* (not shown) among other genes encoding components of the asymmetric cell division machinery. To determine whether Dichaete regulates *pros*, we examined Pros expression in *Dichaete* mutants and observed an increase in Pros levels at stage 10, particularly in the medial column of the neuroectoderm (Figure 5D and E), and by stage 11 Pros was seen at high levels in the neuroectoderm of mutant embryos (Figure 5F and G). Whether this reflects an increase in the number of Pros-expressing cells in the embryo or precocious activation of Pros remains to be determined. By stage 16 Pros levels were considerably reduced compared to wild type (not shown). While it is possible that loss of Pros is due to an indirect consequence of Dichaete loss, our data are consistent with a model where Dichaete is involved in repressing *pros* expression early during neurogenesis but in activating or maintaining *pros* at later stages. Since the expression results could be confounded by indirect effects or by SoxN functional compensation, we examined Pros expression in embryos expressing dominant negative Sox constructs in the neuroectoderm. Whether using Dichaete constructs deleted for the DNA binding domain (ΔHMG, Figure 5I), Engrailed-repressor fusions (Figure 5J) or mouse Sox2 Engrailed-repressor fusions (Figure 5K and L) we found downregulation of Pros in the neuroectoderm from late stage 10 onwards, supporting the view that Dichaete can directly regulate *pros*.

Particularly relevant to the decision to exit the cell cycle to differentiate or undergo apoptosis, we found Dichaete binding at *W, grim* and *rpr* (Figure 5C), indicative of regulatory input into the apoptotic pathway, as well as key cell cycle regulators such as Cyclin E. Combined with our findings relating to the *AS-C* and temporal NB program described above, the binding and expression data indicate that Dichaete plays a key role in the entire pathway from neural specification, through determination of NB identity to the generation of differentiated neural cells.

### The role of Dichaete in the neuroblast regulatory network

**Figure 6:**
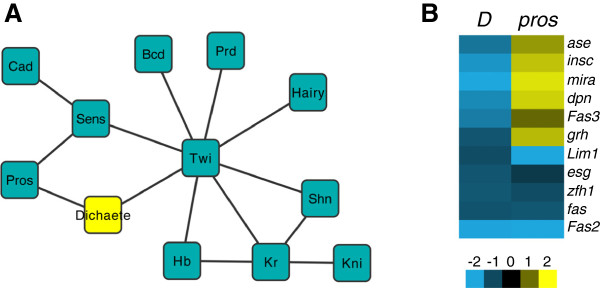
**Dichaete Interactions. A)** Significant binding interval overlaps between pairs of transcription factors generated with Coocur. **B)** Gene expression heatmaps for selected neural genes targets in *Dichaete* and *pros* mutants.

Our analysis of various CNS pathways suggested Dichaete involvement in many different processes and to elaborate this further we compared our core Dichaete binding intervals with a range of binding data generated by the BDTNP and modENCODE projects. We selected binding datasets available for 41 different transcription factors encompassing the first 12 hrs of embryogenesis and used a resampling-based method [57] to identify highly significantly overlapping binding profiles. We performed an exhaustive pair-wise comparison and identified 14 significant overlaps (Figure 6A, Additional file 10: Table S10). Of these, 8 included overlaps with Twist, a mesoderm-specific TF that also shows significant binding at HOT regions in the *Drosophila* genome [58]. We found that Dichaete significantly overlapped with Prospero and Twist. The overlap with Pros is in line with our findings presented above. The overlap with Twist, on the other hand, is unlikely to be of specific functional significance, since there is little overlap between Dichaete and Twist expression during embryogenesis. Rather, the significant overlap may be a reflection of the tendency of both transcription factors to bind at HOT regions.

**Figure 7:**
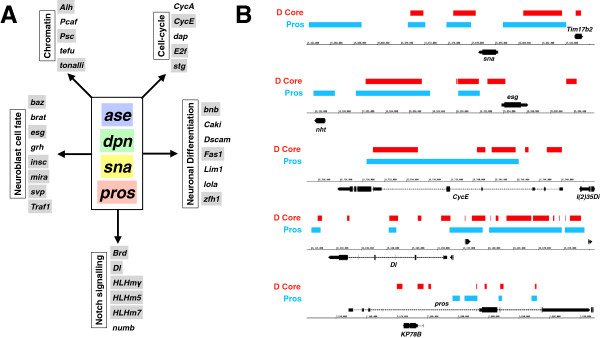
**Dichaete in the neuroblast regulatory network. A)** Representation of the transcriptional regulatory network from [60] is shown. Dichaete target genes (binding and expression change) overlapping with Prospero binding are shaded (coloured for the core genes and grey for downstream functions). **B)** Dichaete and Prospero binding at different genes in the transcriptional regulatory network. Dichaete core intervals are shown in red and *pros-*DamID intervals in blue.

The relationship between Dichaete and Pros was further explored by comparing the Dichaete core binding intervals with Pros-DamID and expression data in more detail [59]. We again found extensive binding overlap, with 902 of 1478 Prospero intervals also Dichaete core intervals. The Prospero/Dichaete overlaps were associated with 704 genes (Additional file 11: Table S11), including 17 genes involved in axon guidance pathways (p = 3.6E-5), as well as a collection of genes involved in the regulation of developmental processes, including cell fate commitment (106 genes, p = 3.1E-39). We also compared gene expression data from *Dichaete* and *pros* mutants and found examples of genes with opposite expression changes (down in *Dichaete*, up in *pros*) as well as genes with similar expression changes (Figure 6B), suggesting Dichaete and Pros may both collaborate and antagonise. Pros promotes neural differentiation and represses self-renewal via a regulatory network involving Ase, Dpn and Sna [60]. We found that Dichaete binding intervals were associated with almost all of the genes in this network (34/35), with over half (20/35) identified as high confidence Dichaete targets (Figure 7A). We also found that 65% (23/35) showed overlapping Dichaete and Prospero binding, including Prospero itself (Figure 7B).

As we note above, Dichaete and mammalian Sox2 show considerable functional similarity and recently, a genomic study identified over ~1400 Sox2 target genes in neural stem cells [61]. We converted this list of mouse genes to their *Drosophila* orthologues (1235 genes) and compared these with genes in the core Dichaete binding set and our list of high confidence direct target genes, identifying 523 and 217 genes respectively (Additional file 12: Table S12 and Additional file 13: Table S13). Over half of the 271 conserved targets are highly enriched for regulation-associated GO terms (P < E-30), 35% annotated as being involved in transcriptional regulation (p = 4.2E-20) and 35% in the generation of neurons (p = 5.7E-30). Remarkably, 13 out of the 34 Dichaete-bound neuroblast regulatory network genes are also Sox2 targets in the mouse neural system, found to be mainly genes connected with Notch signalling (4/6), neuroblast cell fate (3/8) and neuronal differentiation (3/7). This remarkable overlap hints at a deep conservation in the regulatory networks driving the development of neural cells between distantly related species, and points to a key role for group B transcription factors in this process.

## Discussion

The *Drosophila* Dichaete gene encodes a group B Sox domain protein previously implicated in diverse developmental pathways, including key aspects of early segmentation and CNS development [14,15]. We have combined genome-wide binding analysis and gene expression profiling to identify a set of over a thousand high confidence Dichaete targets in the fly genome. Our conservative target list reflects genes bound by Dichaete in independent binding assays and that show very significant expression changes in *Dichaete* mutant embryos. It is likely this represents an underestimate of the true extent of Dichaete activity in the fly genome for several reasons. First, we employed stringent cut-off criteria for both the binding and the gene expression analysis. Second, loss of Dichaete function is frequently compensated by the related Group B protein SoxN, thus many genes showing high confidence binding may not exhibit significant expression changes in *Dichaete* mutants [19,20]. This is supported by recent work expressing dominant negative forms of Dichaete and its vertebrate orthologue Sox2 in the CNS where we identified over 300 additional target genes [21]. In addition, we have recently analysed the genomics of SoxN in the *Drosophila* embryo and find very substantial overlap between Dichaete and SoxN binding as well as a repertoire of genes uniquely bound by each gene (EF and SR, in preparation). Taken together our analysis indicates that Dichaete directly regulates a substantial fraction of genes in the fly genome, particularly in the CNS, suggesting it may act as a regulatory hub. Consistent with this view, we found that over half of the transcription factors encoded in the fly genome are bound by Dichaete and misregulated in the mutant, indicating that Dichaete is likely to be involved in many of the regulatory networks driving CNS development.

The core Dichaete binding intervals we identified are enriched for Sox binding motifs but we also found significant overrepresentation of binding motifs for Vfl, the GAGA-binding factor Trl and the JAK-STAT pathway transcription factor Stat92E. All three of these factors have been identified as key elements in the regulatory programme that drives the onset of zygotic gene expression in the blastoderm embryo [38,62-64]. Dichaete also plays a key role in early zygotic gene expression, regulating the correct expression of pair rule genes [14,15], and we found overlapping Vfl/Dichaete binding at *eve*, *h*, and *run* stripe enhancers. While most of the work on Vfl has focused on understanding its function during the maternal to zygotic transition, the gene is expressed more widely after cellularisation, particularly in the CNS [64]. Indeed recent work has shown a specific role for Vfl in the CNS midline [65], a tissue where Dichaete is known to be active [13,25] and we found overlapping Vfl/Dichaete binding associated with *sli* and *comm*, known Dichaete midline targets. Post cellularisation functions for Trl and Stat92E are well established.

More recently, these three factors, particularly Vfl and Trl, have been strongly associated with enhancer activity driven by Highly Occupied Target (HOT) regions [58]. HOT regions have been identified in large scale studies of the *Drosophila*, *C. elegans* and human genomes, and represent genomic sites where many functionally unrelated transcription factors bind, frequently in the absence of specific binding motifs [66]. The finding that Dichaete binding locations are marked by overrepresentation of binding motifs for factors defining HOT regions, coupled with the widespread gene expression effects of *Dichaete* mutations, suggests that Dichaete may also play a role in regulatory interactions at HOT enhancers. It is notable that Dichaete, in common with all other characterised Sox proteins, is known to bend DNA upon binding [56]. It is possible that Dichaete activity at HOT regions is mediated by this bending activity, helping to bring together complexes of other regulators. In this view, Dichaete would assist binding of factors at non-canonical target sites by favouring protein-protein interactions. In one of the *bona fide* Dichaete regulatory elements that have been studied in detail, the *slit* midline enhancer, Dichaete helps coordinate interactions between the POU factor Vvl and a Sim/Tango heterodimer [25].

Aside from a proposed role at HOT regions, our analysis indicated Dichaete binds to and is active at many characterised regulatory elements. Almost half the enhancers catalogued by RedFly and a substantial fraction of neural enhancers identified by the FlyLight project [45,46] show evidence of Dichaete regulation. Along with this, we observed an association between Dichaete binding and transcriptional start sites, suggesting to us one of two possibilities. Either Dichaete directly engages with core promoter elements or looping interactions between Dichaete bound enhancers and the transcriptional machinery results in ChIP or DamID assays capturing these interactions. In this respect we note that Dichaete binds in the minor groove of DNA, perhaps making it more likely to capture indirect interactions.

Whether Dichaete acts at defined tissue-specific enhancers, HOT regions, core promoters, or all three, our analysis uncovered widespread involvement in specific developmental processes in the embryo. For example, our previous studies highlighted a role for Dichaete in hindgut morphogenesis and identified *dpp* as a likely target gene, since targeted *dpp* expression in the hindgut of *Dichaete* mutants was able to partially rescue the phenotype [16]. Our new analysis implicates Dichaete in the regulation of many of the key factors responsible for hindgut specification and morphogenesis, with most of the characterised transcription factors or signalling pathway components known to be important for hindgut development [52] bound and regulated by Dichaete. This further emphasises the view that Dichaete plays a hub-like role in controlling regulatory networks. We note that hindgut phenotypes and gene expression are unlikely to be functionally compensated by other Sox factors. While the group E gene *Sox100B* is also expressed in the embryonic hindgut [67], we have not seen evidence for synergistic interactions between *Dichaete* and *Sox100B* mutants (SR unpublished observations) and thus functional compensation by Sox100B is less likely. On the other hand, the related group B gene *Sox21b* is expressed in the hindgut and partially overlaps with Dichaete [9]. Although deletions encompassing *Sox21b* show no obvious phenotype, assessing possible functional compensation of Dichaete functions is difficult due to the close proximity of the two genes (~40 kb). It has recently been reported that human SOX2 is involved in gut development where it interacts antagonistically with CDX2 [68]. Caudal is a *Drosophila* orthologue of CDX2 and we found Dichaete binding and associated repression of *cad*, hinting at further levels of regulatory network conservation across metazoa.

In common with vertebrate group B genes, *Dichaete* plays a prominent role in the CNS. Many previous studies focused on single genes have shown that Dichaete is involved in neural specification via the regulation of proneural genes in the Achaete-scute complex [21-23] and our current analysis provides a genomic perspective on this, identifying extensive Dichaete binding across the complex. Importantly, much of this binding coincides with mapped regulatory elements and we found changes in the expression of complex genes in *Dichaete* mutants. Dichaete is involved in the temporal cascade that confers specific identities to neuroblasts and their progeny [32] and our analysis provides considerable insights into this role. We found Dichaete binding associated with all of the characterised genes in the temporal cascade, as well as considerable overlapping binding between Dichaete, Hb and Kr, strongly supporting the idea that cross-regulatory interactions between these genes is important for correct neural specification. For example, maintenance of Hb or loss of Cas, the first and last genes in the cascade, lead to prolonged expression of Dichaete and cells remain in a neuroblast state [32]. Our analysis suggests that Dichaete may help maintain the temporal cascade expression in the neuroblast.

Finally, our analysis uncovered a striking relationship between Dichaete and Pros, with Dichaete negatively regulating *pros* expression early in neural development. In addition, both proteins show an extensive and highly significant overlap in their binding profiles. The gene expression data indicate that Dichaete and Pros may have antagonistic interactions since we find genes encoding neuroblast functions (e.g. *ase*, *insc*, *mira* and *dpn*) are downregulated in *Dichaete* mutants but upregulated in *pros* mutants [59]. However, we also find that genes involved in aspects of neuronal differentiation (e.g. *esc*, *zfh1* and *Lim1*) are positively regulated by both factors. Taken together it is tempting to speculate that in neuroblasts, when Pros is cytoplasmic, Dichaete positively regulates genes required to maintain the self-renewal state and keeps *pros* levels down. In the GMC, Dichaete function must be downregulated to allow cells to exit the cell cycle and differentiate [32], consequently *pros* expression would be upregulated and the protein translocated to the nucleus by the well-established asymmetric division mechanism, repressing neuroblast genes and promoting differentiation. While Dichaete appears to be uniformly expressed in the neuroectoderm, its expression in neuroblasts is dynamic with many neuroblasts expressing Dichaete transiently [23,32]. In addition, and related to the subcellular partitioning of Pros, Dichaete is reported to shuttle between cytoplasm and nucleus, at least early in CNS development [56]. Furthermore, Dichaete is dynamically expressed in GMCs and their progeny, consistent with the proposed interaction with Pros [16]. These observations are consistent with the view that control of Dichaete is important for first determining self-renewal versus differentiation, followed by a role in aspects of neuronal differentiation.

The emerging view from our studies and previous work with Dichaete is of a transcriptional regulator with multifaceted roles in development. We have previously shown that mammalian Sox2 can provide Dichaete function, rescuing *Dichaete* mutant phenotypes. However, the designation of Dichaete as a group B1 protein based on functional arguments is considered by some to be inconsistent with phylogenetic arguments that firmly place Dichaete in the B2 group [11]. In vertebrates, group B2 proteins act as transcriptional repressors, antagonising group B1 functions [6,7]. Since we see very few genes upregulated in *Dichaete* mutants, our analysis suggests that *Dichaete* may be acting primarily as a transcriptional activator. However, this type of mutant expression study is prone to pleiotropic effects, so further investigation of specific targets and tissues is needed. In vertebrates the group B1 proteins play critical roles in the specification and maintenance of neural stem cells, exactly the functions described for Dichaete. The observed correspondence between Dichaete and Sox2 target genes show that these proteins are not only conserved at the functional level when assayed in mutant rescue experiments but also, remarkably, at the level of the gene regulatory networks they control in the fly and mouse nervous system.

One possible explanation for these disparate findings regarding the classification of Dichaete as a group B1 or B2 protein may be provided by the role of Dichaete in the regulation of proneural genes and its early activity on *pros*. In these specific cases, *Dichaete* acts to repress these genes in the neuroectoderm while SoxN acts as an activator [19-22]. It is therefore possible that, in the last common ancestor of the vertebrates and invertebrates when the B1 - B2 split occurred, the ancestral Dichaete gene had an limited B2-like repressor role as well as more prominent B1-like activator role in the CNS. As the lineages diverged the vertebrate B2 genes evolved specialised repressor functions while, in the invertebrates, they maintained more basal activator function. Support for the idea that insect Sox genes represent conserved basal functions of more diverged vertebrate family members comes from experiments replacing the mouse group E gene, *Sox10*, with the fly *Sox100B* coding sequence. In these studies the fly gene is able to provide substantial *Sox10* function in the developing embryo, more so than the *Sox8* gene, which is far closer to *Sox10* at the sequence level [69].

## Conclusions

In summary, we present a rigorous analysis of the genomics of the *Drosophila* group B transcription factor Dichaete, highlighting regulatory input into several key developmental pathways. Our studies provide a baseline for more detailed analysis of highly conserved aspects of group B Sox function in neural stem cells and in neuronal differentiation.

## Methods

### *DamID*

Ecoli Dam was introduced into a pUAST plasmid carrying a full-length Dichaete cDNA to produce a Dam_Dichaete fusion [15] and this vector used to generate transgenic fly lines via standard P-element transformation [70]. A Dam only vector was prepared in parallel. Embryos from homozygous Dichaete-Dam and Dam only lines were collected from 2-7 hrs after egg laying (stages 5–11) and DNA processed for DamID according to the method of Vogel et al. [71]. Biological triplicates of enriched DNA from experimental and control lines were amplified by PCR and Cy3/Cy5 dyes incorporated by random priming with Klenow polymerase. Combined DNA samples were loaded onto Nimblegen D.mel ChIP 2.1 M tiling arrays (GEO platform GPL15057), hybridized overnight at 42°C, then washed and scanned in an Axon GenePIX scanner the following day. Scanned arrays were quantile normalised separately for samples and controls, and peak calling was performed using RINGO [72] to identify binding intervals at different False Discovery Rates. Full DamID data is available from the NCBI Gene Expression Omnibus as part of Super Series GSE49095.

### Gene expression

Stage 10–11 embryos (5 and 7.5 hours after egg laying) from a cross between *D*^*r72*^*/TM3, twi-GAL4 UAS-Gfp D*^*r513*^*/TM3, twi-GAL4 UAS-Gfp*, were hand picked under a fluorescence dissecting microscope. GFP negative homozygous *Dichaete* mutant embryos and their heterozygous single GFP positive siblings were collected and approximately 150 embryos per sample were stored frozen in Trizol. Following RNA extraction, reverse transcription, Klenow amplification and labelling, samples were hybridised to INDAC FL003 (GEO:GPL14121) Drosophila gene expression arrays using our standard protocols (http://www.flychip.org.uk/). Four biological replicates were performed for each experiment, with 2 dye swaps incorporated into the experimental design to control for bias. Arrays were quality checked manually, removing spots affected by high levels of background or artefacts. Our standard data analysis pipeline was employed (http://www.flychip.org.uk/) using Dapple for spotfinding and quantifying signal intensities [73]. The normalisation step was performed using invariant normalisation [50] to address the fact that the majority of genes change expression, with the later analysis stages performed with limma. The thresholds used to find differentially expressed genes were average M-value < −1 or >1, and p-value < 0.01. All gene expression data is available from the NCBI Gene Expression Omnibus as part of Super Series GSE49095.

### Dichaete core binding

Existing ChIP data was obtained from the BDTNP website [34], where we used the published binding intervals, and from modENCODE ([35]; DCCid modENCODE_2571). In the latter case we reprocessed the raw data using the quantile normalisation and peak-calling approaches described above. To create the core dataset, we used intervals supported by at least one low stringency (25% FDR) and one high stringency (1% FDR) dataset from independent experimental techniques. This meant that all intervals confirmed by at least one 1% FDR ChIP and the 25% FDR DamID, and vice versa, were included in the final Dichaete core set (Figure 1A). A .bed file of Dichaete core binding intervals is provided as Additional file 14: Table S14.

### Genome version and annotations

All genomic coordinates are in Genome Release 5 (dm3), and the genome annotations used were FlyBase R5.48, obtained from FlyMine v36.0 [39]. Where relevant, binding coordinates were converted to Genome Release 5 (dm3) using the UCSC liftover tool. Unless indicated otherwise, analysis was performed custom-written Perl scripts (v5.12.4), with the graphs and statistical tests done in R 2.15.2. The gene assignments to intervals were performed as follows: all genes directly overlapping a particular binding interval were assigned as hits to that interval. If an interval was found to overlap no genes, the closest gene would instead be assigned as a hit, up to the maximum of a 10 kb range around both sides of the interval. *Drosophila* orthologues of mouse Sox2 target genes were identified with FlyMine using data from TreeFam release 8.0.

### Gene list analysis

All gene lists were analysed using FlyMine [39] to obtain basic summary statistics and enrichment analysis, including GO term and domain enrichment. For enrichment, FlyMine uses a hypergeometric distribution, and the Holm-Bonferroni correction was applied to get the adjusted p-values. In the case of the binding data, a gene length correction was also applied. The expression heatmap of genes differentially expressed in *Dichaete* null mutants was generated using the Fly Express 6.0 GEMs tool [51]. Data on which genes are likely to be transcription factors was obtained from FlyTF v2 [40].

### Motif analysis

The de novo motif finding was performed using i-CisTarget [37]. The RSAT matrix-scan tool was used for motif scanning: 1^st^ order Markov Model was used to generate the background model from the input sequences, and the sequence matches with the weight score > = 4 were retrieved [74].

### Binding interval location

For the purposes of this analysis, a 1 nucleotide coordinate in the centre of each binding interval was considered to be an approximation of the location of the Dichaete binding event. An overlap with known genome features was then determined using gene, exon and intron coordinates from FlyBase R5.48, obtained via FlyMine. For the transcription start site (TSS) analysis, the start coordinate for each gene was taken as an approximation of the TSS location. Enhancer locations were obtained from the REDFly database version 3.2 [42], and the intervals from the FlyLight project were used as published [45,46]. The overlaps were found using Bedtools v2.17 [75].

### Transcription factor binding overlaps

For the broader analysis of the transcription factor network in embryogenesis, the binding data was not reanalysed. Instead, the peak interval files provided were used for the analysis (a list of the datasets used is provided on Additional file 15: Table S15). In the cases where more than one dataset falling within the 0-12 h period was available for a particular transcription factor, a union of the datasets was created for that TF, and this was used in the co-occurrence analysis [57].

## Competing interests

The authors declare that they have no competing interests.

## Authors’ contributions

JA performed the gene expression experiments and the majority of the data analysis. EF performed the DamID experiments. BF assisted with microarray data analysis. SPS performed the *pros* mutant analysis. SR conceived the study, provided the funding and analysed the data. JA and SR wrote the paper. All authors read and approved the manuscript.

## Supplementary Material

Additional file 1: Table S1 List of genes with Dichaete binding intervals from 1% FDR Dichaete DamID. Click here for file

Additional file 2: Table S2 Gene Ontology Biological Process enrichment analysis of Dichaete-bound genes from 1% FDR DamID. Click here for file

Additional file 3: Table S3 Cooccur Analysis of TF overlaps. Headers: tf1, Dichaete dataset 1; tf2, t Dichaete dataset 1; overlapped_in_tf1, number of TF1 binding interval overlapping TF2; n_tf1, number of binding intervals in TF1 dataset; percent_in_tf1, fraction of TF1 dataset overlapping with TF2; overlapped_in_tf2, number of TF2 binding intervals overlapping with TF1; n_tf2, number of TF2 binding intervals in dataset; percent_in_tf2, fraction of TF2 intervals overlapping TF1; nsamples, number of resampling runs; pvalue, raw p value, padjust; adjusted p value. Click here for file

Additional file 4: Table S4 Core Dichaete bound genes from the amalgamation of DamID and ChIP datasets. Click here for file

Additional file 5: Table S5 Gene Ontology Biological Process enrichment analysis of Core Dichaete-bound genes. Click here for file

Additional file 6: Table S6 RedFly and FlyLight enhancers associated with Dichaete Binding. Click here for file

Additional file 7: Table S7 Genes differentially expressed comparing Dichaete mutant embryos to wild type. Column headers: Transcript, FlyBAse transcript ID; Fbgn, FlyBase gene ID; Gene, FlyBase gene symbol; D_mutant_invar_limma aveM, log2 fold expression change; D_mutant_invar_limma p-value, p-value for expression change. Click here for file

Additional file 8: Table S8 Expression changes of Dichaete target genes. Column headers: FlyBAse transcript ID; Fbgn, FlyBase gene ID; Gene, FlyBase gene symbol; Subsequent columns provide log2 fold expression changes (AveM) and p-values of data from (shen et al. [Ref 21] and from this study, abstracted from Additional file 6: Table S6. Click here for file

Additional file 9: Table S9 Gene Ontology Biological Process enrichment analysis of Dichaete target genes. Click here for file

Additional file 10: Table S10 Cooccur Analysis of TF overlaps. Headers: tf1, transcription factor 1 dataset; tf2, transcription factor 2 dataset; overlapped_in_tf1, number of TF1 binding interval overlapping TF2; n_tf1, number of binding intervals in TF1 dataset; percent_in_tf1, fraction of TF1 dataset overlapping with TF2; overlapped_in_tf2, number of TF2 binding intervals overlapping with TF1; n_tf2, number of TF2 binding intervals in dataset; percent_in_tf2, fraction of TF2 intervals overlapping TF1; nsamples, number of resampling runs; pvalue, raw p value, padjust; adjusted p value; Gene 1, TF 1 gene name; Gene 2, TF2 gene name. Click here for file

Additional file 11: Table S11 Genes bound by Dichaete and Prospero. Click here for file

Additional file 12: Table S12 Drosophila orthologues of mouse Sox2 bound genes in the Dichaete core binding set. Click here for file

Additional file 13: Table S13 Drosophila orthologues of mouse Sox2 bound genes in the Dichaete target gene set. Click here for file

Additional file 14: Table S14 Dichaete Core binding intervals in. bed format. Click here for file

Additional file 15: Table S15 Table of transcription factor binding data used in overlap analysis. Click here for file

## References

[B1] Wegner M Stolt C From stem cells to neurons and glia: a Soxist&apos;s view of neural development Trends Neurosci 2005 28 583 588 10.1016/j.tins.2005.08.008 16139372

[B2] Avilion AA Nicolis SK Pevny LH Perez L Vivian N Lovell-Badge R Multipotent cell lineages in early mouse development depend on SOX2 function Genes Dev 2003 17 126 140 10.1101/gad.224503 12514105 PMC195970

[B3] Bylund M Andersson E Novitch BG Muhr J Vertebrate neurogenesis is counteracted by Sox1–3 activity Nat Neurosci 2003 6 1162 1168 10.1038/nn1131 14517545

[B4] Collignon J Sockanathan S Hacker A Cohen-Tannoudji M Norris D Rastan S Stevanovic M Goodfellow P Lovell-Badge R A comparison of the properties of SOX3 with SRY and two related genes SOX1 and SOX2 Development 1996 122 509 520 8625802 10.1242/dev.122.2.509

[B5] Pevny LH Nicolis SK Sox2 roles in neural stem cells Int J Biochem Cell Biol 2010 42 421 424 10.1016/j.biocel.2009.08.018 19733254

[B6] Uchikawa M Kamachi Y Kondoh H Two distinct subgroups of Group B Sox genes for transcriptional activators and repressors: their expression during embryonic organogenesis of the chicken Mech Dev 1999 84 103 120 10.1016/S0925-4773(99)00083-0 10473124

[B7] Uchikawa M Yoshida M Iwafuchi-Doi M Matsuda K Ishida Y Takemoto T Kondoh H B1 and B2 Sox gene expression during neural plate development in chicken and mouse embryos: Universal versus species-dependent features Dev Growth Differ 2011 53 761 771 10.1111/j.1440-169X.2011.01286.x 21762129

[B8] Cremazy F Berta P Girard F Genome-wide analysis of Sox genes in Drosophila melanogaster Mech Dev 2001 109 371 375 10.1016/S0925-4773(01)00529-9 11731252

[B9] McKimmmie C Woerfel G Russell S Conserved genomic organisation of group B Sox genes in insects BMC Genet 2005 6 26.21 26.15 15943880 10.1186/1471-2156-6-26 PMC1166547

[B10] Wilson MJ Dearden PK Evolution of the insect Sox genes BMC Evol Biol 2008 8 120 10.1186/1471-2148-8-120 18439299 PMC2386450

[B11] Zhong L Wang D Gan X Yang T He S Parallel expansions of Sox transcription factor group B predating the diversifications of the arthropods and Jawed Vertebrates PLoS ONE 2011 6 e16570 10.1371/journal.pone.0016570 21305035 PMC3029401

[B12] Overton P The role of Sox genes in the development of *Drosophila melanogaster* PhD Thesis 2003 University of Cambridge, Department of Genetics

[B13] Sanchez-Soriano N Russell S The Drosophila Sox-domain protein Dichaete is required for the development of the central nervous system midline Development 1998 125 3989 3996 9735360 10.1242/dev.125.20.3989

[B14] Nambu P Nambu J The *Drosophila fishhook* gene encodes a HMG domain protein essential for segmentation and CNS development Development 1996 122 3467 3475 8951062 10.1242/dev.122.11.3467

[B15] Russell SRH Sanchez-Soriano N Wright CR Ashburner M The Dichaete gene of Drosophila melanogaster encodes a SOX-domain protein required for embryonic segmentation Development 1996 122 3669 3676 8951082 10.1242/dev.122.11.3669

[B16] Sanchez-Soriano N Russell S Regulatory mutations of the Drosophila Sox gene Dichaete reveal new functions in embryonic brain and hindgut development Dev Biol 2000 129 1165 1174 10.1006/dbio.2000.9648 10753518

[B17] Overton PM Chia W Buescher M The Drosophila HMG-domain proteins SoxNeuro and Dichaete direct trichome formation via the activation of shavenbaby and the restriction of Wingless pathway activity Development 2007 134 2807 2813 10.1242/dev.02878 17611224

[B18] Cremazy F Berta P Girard F SoxNeuro, a new Drosophila Sox gene expressed in the developing central nervous system Mech Dev 2000 93 215 219 10.1016/S0925-4773(00)00268-9 10781960

[B19] Buescher M Hing FS Chia W Formation of neuroblasts in the embryonic central nervous system of Drosophila melanogaster is controlled by SoxNeuro Development 2002 129 4193 4203 12183372 10.1242/dev.129.18.4193

[B20] Overton P Meadows L Urban J Russell S Evidence for differential and redundant function of the Sox genes Dichaete and SoxN during CNS development in Drosophila Development 2002 129 4219 4228 12183374 10.1242/dev.129.18.4219

[B21] Shen SP Aleksic J Russell S Identifying targets of the Sox domain protein Dichaete in the Drosophila CNS via targeted expression of dominant negative proteins BMC Dev Biol 2013 13 1 10.1186/1471-213X-13-1 23289785 PMC3541953

[B22] Zhao G Boekhoff-Falk G Wilson BA Skeath JB Linking pattern formation to cell-type specification: Dichaete and Ind directly repress achaete gene expression in the Drosophila CNS Proc Natl Acad Sci USA 2007 106 3847 3852 17360441 10.1073/pnas.0611700104 PMC1820672

[B23] Zhao G Skeath J The Sox-domain containing gene Dichaete/fish-hook acts in concert with vnd and ind to regulate cell fate in the Drosophila neuroectoderm Development 2002 129 1165 1174 11874912 10.1242/dev.129.5.1165

[B24] Skeath JB At the nexus between pattern formation and cell-type specification: the generation of individual neuroblast fates in the Drosophila embryonic central nervous system Bioessays 1999 21 1 10 10.1002/(SICI)1521-1878(199901)21:1<1::AID-BIES1>3.0.CO;2-D 10517865

[B25] Ma Y tel KC Gao Y Niemitz E Mosher J Mukherjee A Mutsuddi M Huseinovic N Crews ST Johnson WA Nambu JR Functional interactions between Drosophila bHLH/PAS, Sox, and POU transcription factors regulate CNS midline expression of the slit Gene J Neurosci 2000 20 4596 4605 10.1523/JNEUROSCI.20-12-04596.2000 PMC6772444 10844029

[B26] Ambrosetti DC Basilico C Dailey L Synergistic activation of the fibroblast growth factor 4 enhancer by Sox2 and Oct-3 depends on protein-protein interactions facilitated by a specific spatial arrangement of factor binding sites Mol Cell Biol 1997 17 6321 6329 9343393 10.1128/mcb.17.11.6321 PMC232483

[B27] Archer TC Jin J Casey ES Interaction of Sox1, Sox2, Sox3 and Oct4 during primary neurogenesis Dev Biol 2011 350 429 440 10.1016/j.ydbio.2010.12.013 21147085 PMC3033231

[B28] Masui S Nakatake Y Toyooka Y Shimosato D Yagi R Takahashi K Okochi H Okuda A Matoba R Sharov AA Pluripotency governed by Sox2 via regulation of Oct3/4 expression in mouse embryonic stem cells Nat Cell Biol 2007 9 625 635 10.1038/ncb1589 17515932

[B29] Bery A Martynoga B Guillemot F Joly JS Retaux S Characterization of enhancers active in the mouse embryonic cerebral cortex suggests Sox/Pou cis-regulatory logics and heterogeneity of cortical progenitors Cereb Cortex 2013 10.1093/cercor/bht126 10.1093/cercor/bht126 23720416

[B30] Tanaka S Kamachi Y Tanouchi A Hamada H Jing N Kondoh H Interplay of SOX and POU factors in regulation of the Nestin gene in neural primordial cells Mol Cell Biol 2004 24 8834 8846 10.1128/MCB.24.20.8834-8846.2004 15456859 PMC517870

[B31] Chao AT Jones WM Bejsovec A The HMG-box transcription factor SoxNeuro acts with Tcf to control Wg/Wnt signaling activity Development 2007 134 989 997 10.1242/dev.02796 17267442

[B32] Maurange C Cheng L Gould AP Temporal transcription factors and their targets schedule the end of neural proliferation in Drosophila Cell 2008 133 891 902 10.1016/j.cell.2008.03.034 18510932

[B33] Graham V Khudyakov J Ellis P Pevny L SOX2 functions to maintain neural progenitor identity Neuron 2003 39 749 765 10.1016/S0896-6273(03)00497-5 12948443

[B34] MacArthur S Li XY Li J Brown JB Chu HC Zeng L Grondona BP Hechmer A Simirenko L Keranen SV Developmental roles of 21 Drosophila transcription factors are determined by quantitative differences in binding to an overlapping set of thousands of genomic regions Genome Biol 2009 10 R80 10.1186/gb-2009-10-7-r80 19627575 PMC2728534

[B35] Negre N Brown CD Ma L Bristow CA Miller SW Wagner U Kheradpour P Eaton ML Loriaux P Sealfon R A cis-regulatory map of the Drosophila genome Nature 2011 471 527 531 10.1038/nature09990 21430782 PMC3179250

[B36] van Steensel B Henikoff S Identification of in vivo DNA targets of chromatin proteins using tethered dam methyltransferase Nat Biotechnol 2000 18 424 428 10.1038/74487 10748524

[B37] Herrmann C Van de Sande B Potier D Aerts S i-cisTarget: an integrative genomics method for the prediction of regulatory features and cis-regulatory modules Nucleic Acids Res 2012 40 e114 10.1093/nar/gks543 22718975 PMC3424583

[B38] Liang H-L Nien C-Y Liu H-Y Metzstein MM Kirov N Rushlow C The zinc-finger protein Zelda is a key activator of the early zygotic genome in Drosophila Nature 2008 456 400 403 10.1038/nature07388 18931655 PMC2597674

[B39] Lyne R Smith R Rutherford K Wakeling M Varley A Guillier F Janssens H Ji W McLaren P North P FlyMine: an integrated database for Drosophila and Anopheles genomics Genome Biol 2007 8 R129 10.1186/gb-2007-8-7-r129 17615057 PMC2323218

[B40] Pfreundt U James DP Tweedie S Wilson D Teichmann SA Adryan B FlyTF: improved annotation and enhanced functionality of the Drosophila transcription factor database Nucleic Acids Res 2010 38 D443 D447 10.1093/nar/gkp910 19884132 PMC2808907

[B41] Amit M Donyo M Hollander D Goren A Kim E Gelfman S Lev-Maor G Burstein D Schwartz S Postolsky B Differential GC content between exons and introns establishes distinct strategies of splice-site recognition Cell Rep 2012 1 543 556 10.1016/j.celrep.2012.03.013 22832277

[B42] Gallo SM Gerrard DT Miner D Simich M Des Soye B Bergman CM Halfon MS REDfly v3.0: toward a comprehensive database of transcriptional regulatory elements in Drosophila Nucleic Acids Res 2011 39 D118 D123 10.1093/nar/gkq999 20965965 PMC3013816

[B43] Grosskortenhaus R Pearson B Marusich A Doe C Regulation of temporal identity transitions in neuroblasts Dev Cell 2005 8 193 202 10.1016/j.devcel.2004.11.019 15691761

[B44] Isshiki T Pearson B Holbrook S Doe CQ Drosophila neuroblasts sequentially express transcription factors which specify the temporal identity of their neuronal progeny Cell 2001 106 11 10.1016/s0092-8674(01)00465-2 11525736

[B45] Manning L Heckscher ES Purice MD Roberts J Bennett AL Kroll JR Pollard JL Strader ME Lupton JR Dyukareva AV A resource for manipulating gene expression and analyzing cis-regulatory modules in the Drosophila CNS Cell reports 2012 2 1002 1013 10.1016/j.celrep.2012.09.009 23063363 PMC3523218

[B46] Jenett A Rubin GM Ngo TT Shepherd D Murphy C Dionne H Pfeiffer BD Cavallaro A Hall D Jeter J A GAL4-driver line resource for Drosophila neurobiology Cell Reports 2012 2 991 1001 10.1016/j.celrep.2012.09.011 23063364 PMC3515021

[B47] Noyes MB Meng X Wakabayashi A Sinha S Brodsky MH Wolfe SA A systematic characterization of factors that regulate Drosophila segmentation via a bacterial one-hybrid system Nucleic Acids Res 2008 36 2547 2560 10.1093/nar/gkn048 18332042 PMC2377422

[B48] Goymer P Network biology: why do we need hubs? Nat Rev Genet 2008 9 650 21491638 10.1038/nrg2450

[B49] Zotenko E Mestre J O’Leary DP Przytycka TM Why do hubs in the yeast protein interaction network tend to be essential: reexamining the connection between the network topology and essentiality PLoS Comput Biol 2008 4 e1000140 10.1371/journal.pcbi.1000140 18670624 PMC2467474

[B50] Pradervand S Weber J Thomas J Bueno M Wirapati P Lefort K Dotto GP Harshman K Impact of normalization on miRNA microarray expression profiling RNA 2009 15 493 501 10.1261/rna.1295509 19176604 PMC2657010

[B51] Kumar S Konikoff C Van Emden B Busick C Davis KT Ji S Wu LW Ramos H Brody T Panchanathan S FlyExpress: visual mining of spatiotemporal patterns for genes and publications in Drosophila embryogenesis Bioinformatics 2011 27 3319 3320 10.1093/bioinformatics/btr567 21994220 PMC3223365

[B52] Lengyel JA Iwaki DD It takes guts: the Drosophila hindgut as a model system for organogenesis Dev Biol 2002 243 1 19 10.1006/dbio.2002.0577 11846473

[B53] Arnold CD Gerlach D Stelzer C Boryn LM Rath M Stark A Genome-wide quantitative enhancer activity maps identified by STARR-seq Science 2013 339 1074 1077 10.1126/science.1232542 23328393

[B54] Sanchez-Soriano N In vivo characterisation of the Drosophila Sox gene Dichaete PhD Thesis 1999 University of Cambridge, Department of Genetics

[B55] Wharton KA Jr Crews ST CNS midline enhancers of the Drosophila slit and Toll genes Mech Dev 1993 40 141 154 10.1016/0925-4773(93)90072-6 8494768

[B56] Ma Y Niemitz EL Nambu PA Shan X Sackerson C Fujioka M Goto T Nambu JR Gene regulatory functions of Drosophila Fish-hook, a high mobility group domain Sox protein Mech Dev 1998 73 169 182 10.1016/S0925-4773(98)00050-1 9622621

[B57] Huen DS Russell S On the use of resampling tests for evaluating statistical significance of binding-site co-occurrence BMC Bioinformatics 2010 11 359 10.1186/1471-2105-11-359 20591178 PMC2910723

[B58] Kvon EZ Stampfel G Yanez-Cuna JO Dickson BJ Stark A HOT regions function as patterned developmental enhancers and have a distinct cis-regulatory signature Genes Dev 2012 26 908 913 10.1101/gad.188052.112 22499593 PMC3347788

[B59] Choksi S Southall T Bossing T Edoff K Dewit E Fischer B Vansteensel B Micklem G Brand A Prospero acts as a binary switch between self-renewal and differentiation in Drosophila neural stem cells Dev Cell 2006 11 775 789 10.1016/j.devcel.2006.09.015 17141154

[B60] Southall TD Brand AH Neural stem cell transcriptional networks highlight genes essential for nervous system development EMBO J 2009 28 3799 3807 10.1038/emboj.2009.309 19851284 PMC2770102

[B61] Bergsland M Ramskold D Zaouter C Klum S Sandberg R Muhr J Sequentially acting Sox transcription factors in neural lineage development Genes Dev 2011 25 2453 2464 10.1101/gad.176008.111 22085726 PMC3243056

[B62] Nien CY Liang HL Butcher S Sun Y Fu S Gocha T Kirov N Manak JR Rushlow C Temporal coordination of gene networks by Zelda in the early Drosophila embryo PLoS Genet 2011 7 e1002339 10.1371/journal.pgen.1002339 22028675 PMC3197689

[B63] Harrison MM Li XY Kaplan T Botchan MR Eisen MB Zelda binding in the early Drosophila melanogaster embryo marks regions subsequently activated at the maternal-to-zygotic transition PLoS Genet 2011 7 e1002266 10.1371/journal.pgen.1002266 22028662 PMC3197655

[B64] Tsurumi A Xia F Li J Larson K LaFrance R Li WX STAT is an essential activator of the zygotic genome in the early Drosophila embryo PLoS Genet 2011 7 e1002086 10.1371/journal.pgen.1002086 21637778 PMC3102735

[B65] Pearson JC Watson JD Crews ST Drosophila melanogaster Zelda and Single-minded collaborate to regulate an evolutionarily dynamic CNS midline cell enhancer Dev Biol 2012 366 420 432 10.1016/j.ydbio.2012.04.001 22537497 PMC3358474

[B66] Farley E Levine M HOT DNAs: a novel class of developmental enhancers Genes Dev 2012 26 873 876 10.1101/gad.192583.112 22549952 PMC3347785

[B67] Loh SHY Russell S A Drosophila group E Sox gene is dynamically expressed in the embryonic alimentary canal Mech Dev 2000 93 4 10.1016/s0925-4773(00)00258-6 10781954

[B68] Raghoebir L Biermann K Buscop-van Kempen M Wijnen RM Tibboel D Smits R Rottier RJ Disturbed balance between SOX2 and CDX2 in human vitelline duct anomalies and intestinal duplications Virchows Arch 2013 462 515 522 10.1007/s00428-013-1405-5 23568430

[B69] Cossais F Sock E Hornig J Schreiner S Kellerer S Bosl MR Russell S Wegner M Replacement of mouse Sox10 by the Drosophila ortholog Sox100B provides evidence for co-option of SoxE proteins into vertebrate-specific gene-regulatory networks through altered expression Dev Biol 2010 341 267 281 10.1016/j.ydbio.2010.01.038 20144603

[B70] Karess RE Glover DM P element mediated germ line transformation of *Drosophila* DNA cloning Volume II 1985 Oxford: IRL Press 121 142

[B71] Vogel MJ Peric-Hupkes D van Steensel B Detection of in vivo protein-DNA interactions using DamID in mammalian cells Nat Protoc 2007 2 1467 1478 10.1038/nprot.2007.148 17545983 PMC7032955

[B72] Toedling J Skylar O Krueger T Fischer JJ Sperling S Huber W Ringo–an R/Bioconductor package for analyzing ChIP-chip readouts BMC Bioinformatics 2007 8 221 10.1186/1471-2105-8-221 17594472 PMC1906858

[B73] Buhler J Ideker T Haynor D Dapple: improved techniques for finding spots on DNA microarrays UW CSE Technical Report 2000 UWTR 2000-08-05. http://www.cs.wustl.edu/~jbuhler/dapple/

[B74] Thomas-Chollier M Defrance M Medina-Rivera A Sand O Herrmann C Thieffry D van Helden J RSAT 2011: regulatory sequence analysis tools Nucleic Acids Res 2011 39 W86 W91 10.1093/nar/gkr377 21715389 PMC3125777

[B75] Quinlan AR Hall IM BEDTools: a flexible suite of utilities for comparing genomic features Bioinformatics 2010 26 841 842 10.1093/bioinformatics/btq033 20110278 PMC2832824

